# Kratom-Induced Liver Injury: A Case Series and Clinical Implications

**DOI:** 10.7759/cureus.14679

**Published:** 2021-04-25

**Authors:** Mahesh Botejue, Gurjot Walia, Omar Shahin, Jyotsna Sharma, Rasiq Zackria

**Affiliations:** 1 Graduate Medical Education, Riverside Community Hospital, Riverside, USA; 2 Internal Medicine, University of California (UC) Riverside, Riverside, USA; 3 Internal Medicine, Riverside Community Hospital/University of California (UC) Riverside, Riverside, USA; 4 Internal Medicine, Riverside Community Hospital, Riverside, USA

**Keywords:** dili, kratom, acute liver injury

## Abstract

Drug-induced liver injury (DILI) is among the most common causes of acute liver injury and acute liver failure in the United States. Kratom is an herbal supplement made from the leaves of a tropical evergreen tree (Mitragyna speciosa) that is native to Southeast Asia. Due to its psychotropic and opioid-like activity, there has been an increase in its use as a recreational drug. Despite this increase, little is known regarding the toxicities and adverse effects though it is known to cause DILI. We present two cases of DILI associated with Kratom use.

## Introduction

Drug-induced liver injury (DILI) is one of the most common etiologies of acute liver failure (ALF) in the United States. Patients may present with a range of clinical manifestations from an asymptomatic transaminase elevation to ALF [1-3]. Clinically, DILI has been described as the rise of alanine aminotransferase (ALT) or aspartate aminotransferase (AST) greater than five times the upper limit of normal (ULN), alkaline phosphatase (ALP) greater than two times the ULN, or bilirubin greater than two times the ULN with an associated rise of AST and ALT [4-5]. While certain over-the-counter and prescription medications have been known to cause liver injury, the incidence of herbal and dietary supplements as a precursor has been rising.

## Case presentation

Case 1

A 27-year-old man with no reported medical history presented with sharp, non-radiating, epigastric abdominal pain, which started one day earlier. He denied any inciting event and reported the pain had been constant since onset. Associated symptoms included fevers, chills, and nausea; he denied vomiting or changes in bowel habits. He denied any alcohol or drug abuse. He did, however, endorse a two-week history of taking Kratom for foot osteoarthritis. Physical examination was unremarkable on presentation except for abdominal pain on palpation. Initial lab work demonstrated elevated liver chemistries of AST 345 U/L, ALT 404 U/L, ALP 115 U/L, and total bilirubin of 1.3 mg/dL. His liver synthetic function tests were normal. A urine drug screen was positive for opiates and tetrahydrocannabinol. Infectious workup, autoimmune panel, salicylate, and acetaminophen toxicity were negative. Imaging studies, including a right upper quadrant ultrasound (US) and magnetic resonance cholangiopancreatography (MRCP), were unremarkable. He was treated symptomatically for DILI from Kratom use. His liver chemistries down-trended with a resolution of his symptoms, and he was discharged home in stable condition.

Case 2

A 36-year-old woman, with a history of hepatic steatosis, ventral hernia repair complicated by two episodes of dehiscence, opioid dependence, and recent choledocholithiasis status post biliary stent placement five months prior, with subsequent removal one month ago and cholecystectomy, presented for worsening jaundice of one-month duration. She reported the onset of symptoms after biliary stent removal and also endorsed lethargy, poor appetite, non-bilious vomiting, and watery diarrhea. She denied any medication, alcohol, or drug use. On presentation, physical examination demonstrated jaundice, scleral icterus, moderate ascites with a positive fluid wave, and pitting edema. Lab work revealed leukocytosis 16.8 K/mm^3^, sodium 132 mmol/L, hyperbilirubinemia 39.5 mg/dL, AST 138 U/L, ALT 28 U/L, ALP 156 U/L, international normalized ratio (INR) of 2.7, and albumin of 2.3 g/dL. Other lab studies included an unremarkable autoimmune and hepatitis panel. The US demonstrated fatty liver with no bile duct dilatation. MRCP revealed hepatomegaly with diffuse fatty infiltration without biliary tree dilatation. Given no identifiable biliary or hepatocellular etiology, a core liver biopsy demonstrated cholestatic hepatitis consistent with DILI, and steatohepatitis with marked pericellular fibrosis and scarring. On further questioning, she endorsed using an herbal supplement, Kratom, for several years to aid with her opioid abuse and withdrawal. Due to a persistently poor model for end-stage liver disease (MELD)-Na score, she was transferred to a liver transplant center for appropriate management.

## Discussion

Kratom, known by many street names, including “thang,” is available in several forms, including powder, tea, extract, tablets, or capsules. It behaves as a stimulant at low doses, opioid-like at moderate doses, and sedative at high doses [6-9]. With the recent promulgation of over-the-counter supplements, physicians must be familiar with their various forms, uses, and toxicities.

In the US, poison centers have reported an increasing number of calls regarding exposure to Kratom [10]. A literature review studied the cases of DILI in the Drug-Induced Liver Injury Network and found eight out of 404 cases to be associated with Kratom [11]. This review determined a causal association in seven out of eight of those cases, with the median Kratom use of 22 days before the onset of injury and reported symptoms of jaundice, itching, abdominal pain, or fever. Of these, all patients recovered, with six hospitalized. This herbal substance is currently considered a “drug of concern” by the Drug Enforcement Administration (DEA), which initially was to classify the extract as a Schedule I drug, but the classification was postponed [12].

Although the hepatotoxicity of Kratom has been suggested, it is unclear to what extent any byproducts or contaminants may have a role in liver injury. A recent comprehensive review discussed the mechanisms of associated hepatotoxicity and suggested that physiologic metabolism is likely hepatic [13]. However, case reports have demonstrated a cholestatic or mixed pattern as well [14].

One of the more likely explanations for the increasing number of Kratom-associated DILI is the opioid epidemic and policymaking shift, including Centers for Disease Control and Prevention (CDC) guidelines, which discourage physicians from prescribing opioids for non-cancer pain [15]. A majority of Kratom users reported using the drug as a treatment for acute or chronic pain, endorsed fewer side effects, and less addiction potential than with opioids [16]. As chronic pain and opioid prescription patterns evolve, this has the potential to encourage the use of less regulated compounds as both an adjunct and replacement for traditional pain control methods [17].

## Conclusions

These cases highlight a rare but serious complication of DILI associated with Kratom use. This contributes to the growing body of evidence supporting Kratom as a cause of liver injury. A thorough history of medication and supplement use is critical for patients with hepatic dysfunction or possible suspicion of DILI.
